# Neural Correlates of Task-Irrelevant First and Second Language Emotion Words – Evidence from the Emotional Face–Word Stroop Task

**DOI:** 10.3389/fpsyg.2016.01672

**Published:** 2016-11-01

**Authors:** Lin Fan, Qiang Xu, Xiaoxi Wang, Feng Zhang, Yaping Yang, Xiaoping Liu

**Affiliations:** ^1^National Research Centre for Foreign Language Education, Beijing Foreign Studies UniversityBeijing, China; ^2^Department of Psychology, Ningbo UniversityNingbo, China; ^3^College of Foreign Language and Literature, Ningbo UniversityNingbo, China; ^4^School of English Studies, Tianjin Foreign Studies UniversityTianjin, China

**Keywords:** emotion word, emotionality, emotional conflict control, bilingualism, emotional Stroop, emotional face–word Stroop

## Abstract

Emotionally valenced words have thus far not been empirically examined in a bilingual population with the emotional face–word Stroop paradigm. Chinese-English bilinguals were asked to identify the facial expressions of emotion with their first (L1) or second (L2) language task-irrelevant emotion words superimposed on the face pictures. We attempted to examine how the emotional content of words modulated behavioral performance and cerebral functioning in the bilinguals’ two languages. The results indicated that there were significant congruency effects for both L1 and L2 emotion words, and that identifiable differences in the magnitude of the Stroop effect between the two languages were also observed, suggesting L1 is more capable of activating the emotional response to word stimuli. For event-related potentials data, an N350–550 effect was observed only in the L1 task with greater negativity for incongruent than congruent trials. The size of the N350–550 effect differed across languages, whereas no identifiable language distinction was observed in the effect of conflict slow potential (conflict SP). Finally, more pronounced negative amplitude at 230–330 ms was observed in L1 than in L2, but only for incongruent trials. This negativity, likened to an orthographic decoding N250, may reflect the extent of attention to emotion word processing at word-form level, while the N350–550 reflects a complicated set of processes in the conflict processing. Overall, the face–word congruency effect has reflected identifiable language distinction at 230–330 and 350-550 ms, which provides supporting evidence for the theoretical proposals assuming attenuated emotionality of L2 processing.

## Introduction

The relationship between affective processing and bilingualism is complex (Kazanas and Altarriba, 2016, p. 396; Pavlenko, 2012). The past decade has witnessed a great surge of interest in the interdisciplinary inquiry of bilingualism and emotion. Implications of bilingualism on emotions have been investigated through diverse methodologies, such as the clinical (e.g., Santiago-Rivera and Altarriba, 2002), introspective (e.g., Pavlenko, 2005; Dewaele, 2010), cognitive (e.g., Eilola et al., 2007; Sutton et al., 2007), psychophysiological (e.g., Harris, 2004; Caldwell-Harris and Ayçiçegi-Dinn, 2009) and electrophysiological (Conrad et al., 2011; Opitz and Degner, 2012) approaches. A majority of studies have pointed to an assumption that L2 affective processing may be characterized by attenuated emotionality compared to L1 (Pavlenko, 2012, for a review).

However, there were discrepancies over the empirical evidence on the emotionality of the bilinguals’ two languages using a variety of tasks such as recall, rating and recognition tasks (e.g., Anooshian and Hertel, 1994; Ayçiçegi and Harris, 2004). Some studies have revealed identifiable differences between the processing of L1 and L2 emotional information (e.g., Anooshian and Hertel, 1994; Altarriba, 2003; Harris et al., 2003). Other researchers have found that emotionally charged entries elicited similar skin conductance levels (SCLs) in L1 and L2 for early bilinguals, and that similar patterns of activation were observed for all the other categories of emotion words except reprimands for late bilinguals (Harris et al., 2006). Likewise, researchers have found that late German-Greek bilinguals were equally capable of automatically activating motor response to emotion word stimuli in the two languages even when bilinguals acquired their L2 rather late in life (age > 11 years old) (Dudschig et al., 2014).

The emotional Stroop task, a variant of the classic color Stroop interference paradigm (Stroop, 1935) has also been adapted to investigate the processing and representation of bilingual emotional words, and this line of research is more closely related to the present study as it also explores the processing of emotion words with Stroop task. In the bilingual version of emotional Stroop task, participants are shown emotional or non-emotional words instead of congruent (GREEN in green) and incongruent (GREEN in red) stimuli in both languages, and they are asked to identify the print color of the words presented. It has been demonstrated that emotionally charged words (e.g.,“fear”, “delight”) produced longer response latency (i.e., more interference) relative to neutral words (e.g., “boat”, “table”). This emotional interference effect shows that more attentional resources are allocated towards the emotionally salient information (Williams et al., 1997), and is a result of the emotional content of the L1 and L2 words and not the congruency of the words presented (cf. Sutton et al., 2007). Employing the emotional Stroop paradigm, Eilola et al. (2007) and Sutton et al. (2007) examined the automatic access of emotional information in early L2-dominant Spanish-English bilinguals and late Finish-English bilinguals, and they found the same amount of interference in the bilinguals’ two languages with negatively valenced emotion words and neutral control words. Grabovac and Pléh (2014) compared the early Hungarian-Serbian bilinguals’ activation of emotionally charged words and neutral words, and similar pattern of interference was observed in their two languages. Contradictory research findings have also been reported in the emotional Stroop task in bilinguals. For instance, studies with late Thai-English bilinguals revealed identifiable differences between the automatic activation of emotional content in L1 and L2 emotion words (Winskel, 2013). Furthermore, mixed results were observed within one single study with empirical evidence from different tasks. Eilola and Havelka’s (2011) emotional Stroop experiment indicated that the behavioral responses showed no significant difference between the emotional activation of participants’ L1 (English) and L2 (Greek) emotion words, but the SCLs indicated a reliable difference between the activation of the two languages in the condition of emotion and taboo words.

The overview of prior research showed that there were contradictory research findings on the automatic activation of emotional information in bilinguals’ two languages across a number of methodological manipulations. The discrepancy may be attributed to a number of differences between studies, such as the tasks administered and the linguistic materials employed, as well as the relative proficiency levels and AoA (age of acquisition) of the bilingual sample (cf. Winskel, 2013). In addition, some researchers argued that the Stroop effect is not defined in the emotional Stroop task (Algom et al., 2004). Therefore, an alternative paradigm, a correspondingly emotional version of the color-word Stroop task, the emotional face–word Stroop tasks could be applied to further investigate the emotionality of L1 and L2 emotion word processing with bilingual speakers, thus providing more empirical evidence to unveil the automatic activation of emotional information processing in the two languages of the bilingual mind. This paradigm provides an automatic measure of word stimuli with emotional information and has been adopted in a number of studies to explore the automatic activation of emotional content in L1 words (e.g., Anes and Kruer, 2004; Chechko et al., 2013). In this task, the emotion word and the face emotion are semantically related, and the response activation from an emotion word would compete with the one from the face emotion.

Furthermore, in the field of cognitive neuroscience, the high temporal resolution of event-related potential (ERP) techniques offer a promising means to explore the neural activity of the overall consecutive stages of information processing from stimulus- to response-onset at the millisecond level (Fabiani et al., 2007; Caldas et al., 2012). The effects of emotion valence on word processing have been extensively examined in native language processing (e.g., Kissler et al., 2006, 2007, 2009; Herbert et al., 2008; Schacht and Sommer, 2009; Scott et al., 2009). Two studies contrasted the ERPs of L1 and L2 emotion word processing (Conrad et al., 2011; Opitz and Degner, 2012), and found that the early posterior negativity (EPN) effect of the bilinguals’ two languages did not qualitatively differ, though it was delayed for L2. In particular, color-word Stroop studies using ERPs to elucidate neural correlate of robust Stroop effect have consistently reported two specific ERP components, which have been mediated by task-relevant and task-irrelevant contingency (Liotti et al., 2000; West and Alain, 2000; West, 2003; Larson et al., 2009, 2014). The medial frontal negativity (MFN) or alternatively N450 (West, 2003; Larson et al., 2014) reflects greater negativity for incongruent than congruent trials with a latency of about 300-500 ms at fronto-central sites (Liotti et al., 2000; Perlstein et al., 2006). There has been evidence that N450 is associated with the conflict processing activity of the anterior cingulate cortex (ACC) (Liotti et al., 2000; West, 2003). With an enhanced positivity for incongruent than congruent trials at centro-parietal electrode sites, the conflict SP starts approximately 500 ms after stimulus onset (Larson et al., 2009, 2014; Naylor et al., 2012). Evidence reveals that conflict SP is related to a set of cognitive processes such as conflict resolution and adaption (Naylor et al., 2012; Larson et al., 2014). In fact, these two ERP components are not only elicited in the classic color-word Stroop task, but also in a variety of Stroop variants, including the emotional face–word Stroop task, which might suggest that these ERPs be associated with the conflict processing in general (Chen et al., 2011). For instance, Shen et al. (2013) observed increased negativity for incongruent than for congruent trials at the time widow of 350-550 ms (N350–550) and enhanced positivity for incongruent than for congruent trials at the time window of 700-800 ms (conflict SP). A similar pattern of results was also obtained in Xue et al. (2015) using the same emotional face–word Stroop task. Three other studies also employed the emotional face–word Stroop paradigm to examine the neural correlates of emotional conflict in L1 processing (i.e., Zhu et al., 2010; Baggott et al., 2011; Zhu and Luo, 2012). Zhu et al. (2010) demonstrated that the enhancement of task-relevant information and suppression of task-irrelevant information began at an early perceptual processing stage, as indexed by N170 component. Likewise, Baggott et al. (2011) found that emotional congruency of word-face pairs affected N170 response. Zhu and Luo (2012) demonstrated that the processing of emotional content in words might have no influence on the facial expression processing in 60-90 ms early time window with C1 component, as evidenced by significant differentiation between fearful and happy faces regardless of emotional congruency of trials. For these emotional face–word Stroop studies among Chinese native speakers (i.e., Zhu et al., 2010; Zhu and Luo, 2012; Shen et al., 2013; Xue et al., 2015), two Chinese emotion words (i.e., “
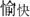
”/“happy” and “
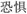
”/“fearful”) and an equal number (20) of Chinese emotional face pictures was employed as experimental stimuli to form face–word pairs that were congruent or incongruent. The current study attempts to further elucidate the generality of prior research findings in L1 using a larger set of emotion word stimuli in both languages of the Chinese-English bilinguals (12 emotion words for each language).

Prior analyses of Stroop and Stroop-like tasks on the emotionality of L1 and L2 emotion words rely heavily on behavioral evidence either pointing to the attenuated emotionality in L2 as compared to L1 (e.g., Eilola and Havelka, 2011; Winskel, 2013) or the same amount of interference in the activation of emotional information in the bilinguals’ two languages (Eilola et al., 2007; Grabovac and Pléh, 2014). However, studies intending to directly contrast ERPs for the bilinguals’ emotion word processing in their two languages are scant. Two ERPs studies have addressed the emotionality in L1 and L2 (i.e., Conrad et al., 2011; Opitz and Degner, 2012), yet these studies revealed no qualitative differences regarding the differential effects as a function of language. Furthermore, no study to date has been conducted to compare the neural correlates of L1 and L2 emotion words with Stroop or Stroop-like tasks.

To further understand the mechanisms underlying the activation and the representation of emotion words in the bilingual lexicon, the emotional face–word Stroop task was applied to tap into the automatic lexical access to late Chinese-English bilinguals’ two languages. To date, there was no investigation on the access to affective valence of L1 and L2 words in the task-irrelevant dimension with the face evaluation task of the emotional face–word Stroop paradigm. This study, to our knowledge, represents the first attempt to unveil the processing of L1 and L2 emotionally charged lexical entries with the emotional face–word Stroop paradigm. Using the high temporal resolution of ERPs, the present study aims to investigate the automatic activation of emotion word across languages, by examining cross-linguistic differences in the mechanism underlying the Stroop interference effect in bilingual population with both behavioral and ERP evidence. Based on previous studies, we hypothesize that Chinese and English tasks in the present work would elicit significant N350–550 and conflict SP, and that the magnitude of interference would be larger in the L1 than in the L2 task.

## Materials and Methods

### Participants

Twenty-three (11 females and 12 males) native speakers of Chinese participated in the experiment. They were undergraduate students from Ningbo University, and all of them passed TEM-4 (Test for English Majors Band 4) (lowest band of TEM) with an average score of 75 out of 100. TEM-4 is a nationwide standardized proficiency test, organized by the Ministry of Education of the People’s Republic of China and administrated only in China. Our participants were all strongly right-handed (handedness: right = 11.21, left = 0.82), as assessed by the Edinburgh Inventory (Oldfield, 1971) and have normal or corrected-to-normal vision with no history of neurological or psychiatric impairment. Participants all signed informed consent and were paid for their participation. The study was under the permission of the Ningbo University Ethics Committee. Among them, one has been to an English-speaking country for six months as an exchange student.

Prior to the formal experiment, we administered the Language Experience and Proficiency Questionnaire (LEAP-Q) (Marian et al., 2007) to the participants to assess their language profiles. As revealed by the received data, the participants’ average AoA of English (L2) is 10.9 years old and they have learned Chinese (L1) since their birth. AoA can predict language proficiency level in a highly reliable manner. Normally, participants with an average AoA of two languages before 5 years old are defined as early bilinguals (Chan et al., 2008; Grossi et al., 2010), and those with an average AoA after 10 years old (at puberty or after puberty) are defined as late bilinguals (Grossi et al., 2010; Hsu et al., 2015). Therefore, participants in the current study can be identified as late bilinguals. Moreover, they have similar language exposure and cultural background according to the received information from LEAP-Q in terms of the context of acquisition (CoA) of their two languages. The results of participants’ self-reported proficiency in their two languages on a 7-point scale indicated significant differences between Chinese (*M* = 6.82, *SD* = 0.51) and English (*M* = 4.94, *SD* = 0.63), *t* = 16.71, *p* < 0.001.

### Stimuli

Compound stimuli which consisted of face pictures and emotion words were used in this experiment. Ten fearful and ten happy face pictures were selected from CAPS (Chinese affective picture system) (Bai et al., 2005), with face pictures of each valence comprising five male and five female faces, respectively. There were two sets of word stimuli. The first set consisted of six positively valenced English emotion words and an equal number of emotionally identical two-character Chinese counterparts. The second set contained six negatively valenced English emotion words together with six emotionally identical two-character Chinese counterparts. For instance, “delight” and “
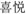
”; “fright” and “
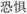
” (**Table 1**). L2 stimulus words were selected from the BNC (British National Corpus), and no significant differences were found in terms of word frequency [*t*(10) = -0.27, *p* = 0.795], syllables [*t*(10) = 0.54, *p* = 0.599) and word length [*t*(10) = -0.43, *p* = 0.673] between the negatively and positively valenced L2 words. In addition, there were no statistical differences between L1 negative and positive words in terms of their frequency [*t*(10) = 1.21, *p* = 0.255] and strokes [*t*(10) = -1.76, *p* = 0.110]. The frequencies of L1 words were taken from *A Dictionary of the Frequency of Commonly Used Modern Chinese Words (Alphabetical sequence section)* (Liu et al., 1990), a 25-million word collection; L2 word frequencies were taken from BNC, a 100-million word database of written and spoken word tokens^1^. To compare the frequency of words from two separate databases, the databases involved should be converted into the same size using the following conversion procedure (conversion formula: 25,000,000/100,000,000 = current frequency of L2 words/original frequency of L2 words). No significant difference was observed between the frequencies of L1 stimulus words and that of L2 stimuli [*t*(22) = 0.33, *p* = 0.744].

**Table 1:** List of word stimuli.

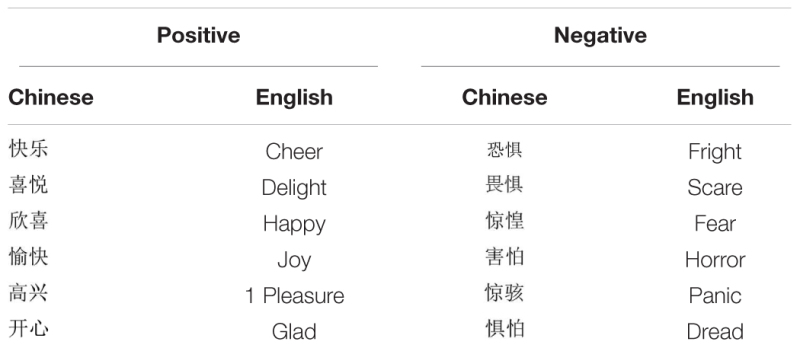

**FIGURE 1:**
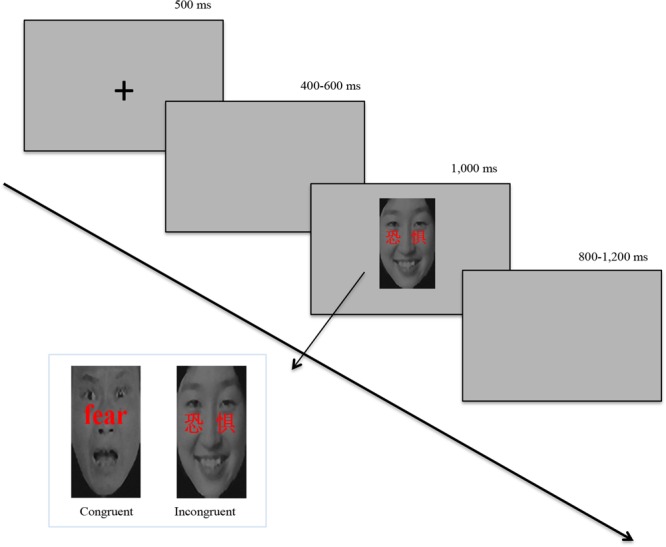
**Flowchart of one experimental trial and compound stimuli samples.** Each trial begins with the presentation of a fixation (“+”, 500 ms, with a blank screen ISI, randomly 400—600 ms duration), followed by the combined stimuli (3.5° wide, 5° high) appearing in the center of the screen (time limit: 1,000 ms, with a blank screen ITI, randomly 800-1,200 ms duration). When participant’s response time is shorter than programmed time limit (1,000 ms), presentation time equals participant’s response time. When participant’s response time is longer than programmed time limit (1,000 ms), presentation time is 1,000 ms.

Four hundred and eighty compound stimuli were prepared with 24 negatively or positively valenced words in prominent red color superimposed on 20 happy or fearful face pictures. The word and facial expression of a compound stimuli was either congruent (e.g., “delight”/“
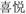
” was superimposed onto a happy face picture) or incongruent (e.g., “delight”/“
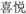
” was superimposed onto a fearful face picture) (see **Figure 1**).

### Procedure

Participants were seated in a quiet room, and faced a monitor placed at 100 cm distance from the eyes. They were asked to respond as quickly and accurately as possible to the facial expressions of emotion by pressing a button corresponding to fearful (left index finger) or happy expression (right index finger). Following the paradigm of previous studies (Zhu et al., 2010; Shen et al., 2013; Xue et al., 2015), the presentation of a fixation “+” for 500 ms, was followed by a blank screen ISI for a randomized duration of 400 or 600 ms. We considered this time interval could help the participants to get better prepared for the face–word compound stimuli presented in the screen. Then, the face–word compound stimuli (3.5° wide, 5° high) appeared in the center of the screen, which was presented for 1,000 ms, and was followed by a blank screen ITI for a randomized duration of 800 or 1,200 ms. When participants’ response time (RT) was shorter than programmed time limit (1,000 ms), presentation time equaled participants’ response latency. When the RT was longer than the programmed time limit (1,000 ms), presentation time was 1,000 ms, and there was an additional 500 ms for them to make a response (see **Figure 1**).

E-Prime 2.0 software package (Psychological Software Tools, Pittsburgh, PA, USA) was used for the stimulus presentation and data acquisition. Participants performed two versions of tasks, one in Chinese and the other in English. Each version comprised 240 compound stimuli, half congruent and half incongruent, which were randomized. There were eight blocks in total, each consisting of 30 compound stimuli. There was a customized recess between every two blocks. Half of the participants performed L1 task first, and the other half performed L2 tasks first, and they were randomly assigned to this arrangement. All participants took part in a practice session during which they were familiarized with the tasks in both languages and the procedures of the experiment.

### EEG Recording

Brain electrical activity was continuously recorded with Neuroscan Aynamp^2^ Amplifier using a 64-channel Ag/AgCl electrode cap (NeuroScan Inc., Herndon, VA, USA), including two mastoid electrodes and four eye movement electrodes (two at the external canthi and two infreorbital), all of which are referenced to the electrode placed on the tip of the nose (band pass 0.05-80 Hz, sampling rate 500 Hz/channel, impedance <5 kΩ). Eye movement artifacts (blinks and eye movement) were rejected oﬄine using the method proposed by Semlitsch et al. (1986).

The ERPs were time-locked with the onset of the face–word combined stimuli. Separate EEG epochs of 1,200 ms (200 ms pre-stimulus and 1000 ms post-stimulus) were extracted oﬄine for all channels. And the length of the baseline was 200 ms between -200 ms and 0 ms before the stimuli onset. Only the ERPs elicited by the correct facial emotion judgments were included in averaging, and trials contaminated by amplifier clipping, bursts of electromyographic activity, or peak-to-peak deflection exceeding ±100 μv were excluded from averaging. ERPs were selectively averaged for each subject and for four conditions: L1-Congruent, L1-Incongruent, L2-Congruent, and L2-Incongruent (>70 trials). The averaged ERPs were digitally filtered with a low-pass filter at 30 Hz (24dB/octave). Grand averages of waveforms at specific electrodes across all participants for each condition were computed as well.

Our main interest of ERP effects was related to the contrasts of the Stroop effect across L1 and L2. Inspection of the grand-average waveforms for Congruent and Incongruent ERP waves and for the Incongruent minus Congruent ERP difference waves in L1 and L2 conditions, as well as their sequential topographical maps of waves, suggested that there might be three major effects that were associated with L1-L2 difference and Stroop related changes: an early one (230–330 ms), a second one (350-550 ms) and a late one (700-900 ms).

The early effect was investigated from 230 to 330 ms on the initial waves. According to the topographical maps and based on previous studies about scalp distribution concerning the N250 component, three midline sites along the anterior-posterior axis (F_Z_, FC_Z_, C_Z_) and four adjacent lateral sites were selected (F1, FC1, C1; F2, FC2, C2). For each condition, repeated-measures ANOVAs were conducted with *Congruency* (Congruent vs. Incongruent), *Language* (L1 vs. L2), as within-subject variables.

The second effect was explored in 100 ms time windows from 350-550 ms on the Incongruent minus Congruent difference waves. Our analysis of the second effect included the frontal (F3, F_Z_, and F4), the frontal-central (FC3, FC_Z_, and FC4), the central (C3, C_Z_, and C4), and the parietal (P3, P_Z_, and P4) electrodes. The scalp distribution is basically in accord with previous findings for N450 (e.g., Liotti et al., 2000; Shen et al., 2013). Repeated-measures ANOVAs were conducted with the factor being *Congruency, Language*.

The last effect was explored in 100 ms time windows from 700 to 900 ms. Based on the topographical maps in each time window (700-800, 800-900 ms) and the scalp distribution reported in previous studies (e.g., Liotti et al., 2000; Shen et al., 2013), a repeated-measures ANOVA to explore the effect of congruency and language was conducted with three electrodes sites on each hemisphere, consisting of a central site (C1, C_Z_, and C2), a central-parietal site (CP1, CP_Z_, and CP2), and a parietal site (P1, PZ, and P2). For all analyses, *p*-values below 0.05 were considered significant, corrected by means of the Greenhouse-Geisser epsilon (Greenhouse and Geisser, 1959).

## Results

### Behavioral Performance

We were unable to use the data from four participants due to insufficient artifact-free trials (<70) and excessive movement. One female participant was excluded from the data analysis due to her exceptionally high error rate (49.8%). This left us with a total of 18 participants for the final analysis (7 females, mean age = 22.33 years, *SD* = 0.84). For each participant in each condition, items with RT beyond 3 SD away from the mean were excluded. This procedure resulted in an average data loss of 1.37%. Only correct responses were included in the analyses of the RT data. Participants’ mean RT for congruent and incongruent conditions and Stroop interference effect (Incongruent minus Congruent difference) in each task was presented in **Table 2**.

**Table 2:** Mean reaction time (ms) and accuracy rate (%) with standard deviation (SD) in parentheses.

Task	Chinese Congruent	Chinese Incongruent	English Congruent	English Incongruent	Stroop^a^ interference (Chinese)	Stroop^a^ interference (English)
Mean RT	555.44 (50.49)	602.56 (65.39)	566.56 (63.46)	588.72 (67.49)	47.11 (24.97)	21.16 (18)
Accuracy	96.3 (3.7)	89.6 (4.5)	94.7 (5.3)	91.7 (5.2)		

*^a^Stroop interference reported here was the Incongruent minus Congruent difference.*

A series of ANOVAs were conducted to examine the main effect of *congruency* (Congruent vs. Incongruent trials) as well as the interaction between *Congruency* and *Language* (Congruency vs. L1 and Congruency vs. L2). For RT, the main effect of congruency was significant [*F*(1,17) = 73.13, *p* < 0.001, η_p_^2^ = 0.81], with RT for congruent trials being shorter than that for incongruent trials. Complementing this finding, significant interaction between *Congruency* × *Language* was found [*F*(1,17) = 16.38, *p* = 0.001, η_p_^2^ = 0.49], suggesting that L1 Stroop effect (*M* = 47.11 ms) was significantly larger than L2 Stroop effect (*M* = 22.17 ms). Simple effect tests probing this interaction revealed a robust congruency effect in L1 [*F*(1,17) = 64.10, *p* < 0.001, η_p_^2^ = 0.79] and L2 tasks [*F*(1,17) = 28.60, *p* < 0.001, η_p_^2^ = 0.63].

Consistent with the results of response latency, the main effect of congruency was significant for response accuracy [*F*(1,17) = 86.56, *p* < 0.001, η_p_^2^ = 0.84], with accuracy rate for congruent trials being higher than that for incongruent ones. In addition, the *Congruency* × *Language* interaction was significant [*F*(1,17) = 14.42, *p =* 0.001, η_p_^2^ = 0.46], suggesting an increased congruency effect in L1 relative to L2 tasks. Simple effect tests revealed that such congruency effect prevailed in L1 [*F*(1,17) = 96.76, *p* < 0.001, η_p_^2^ = 0.85] and L2 tasks [*F*(1,17) = 16.21, *p* = 0.001, η_p_^2^ = 0.49].

### ERPs Results

**FIGURE 2:**
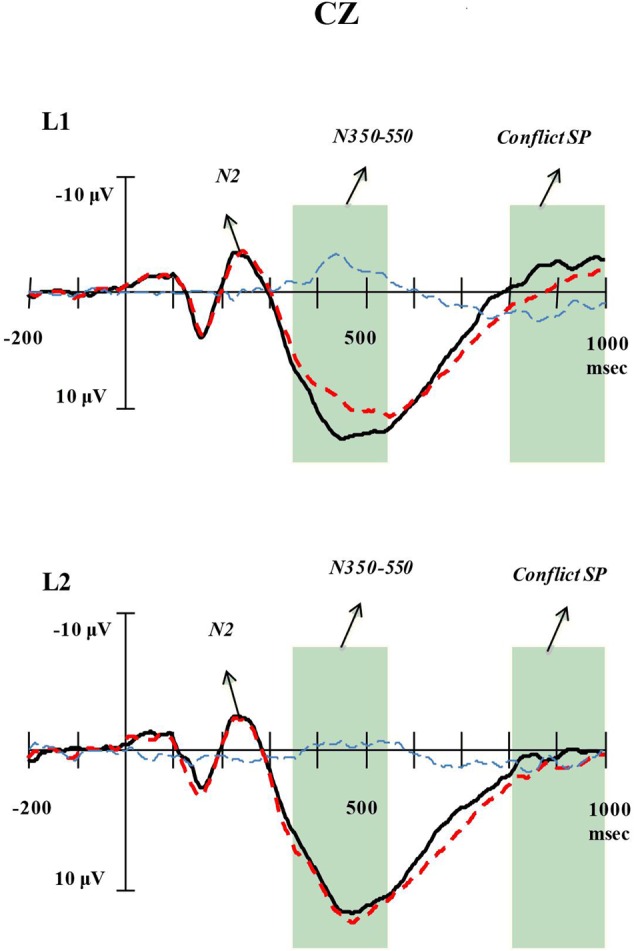
**Detailed information of each component at C_Z_ Site.** Grand-average ERPs at C_Z_ electrode for congruent (Con) emotion words (black line) and incongruent (InC) emotion words (red line), as well as the incongruent minus congruent emotion words difference wave (blue line) for L1 and L2 tasks.

Response times that exceeded 3 SD above or below the mean for each participant were removed from data analysis of ERP data (less than 1.41%). Repeated measures ANOVAs were performed to quantify overall main effects and interactions, with Congruency (congruent, incongruent), Language (L1, L2) as within-subjects variables. Detailed information about each component in L1 and L2 tasks was presented in grand-average waveforms at C_Z_ Site for Congruent and Incongruent ERP waves (**Figure 2**).

### Conflict-Related N350–550

**FIGURE 3:**
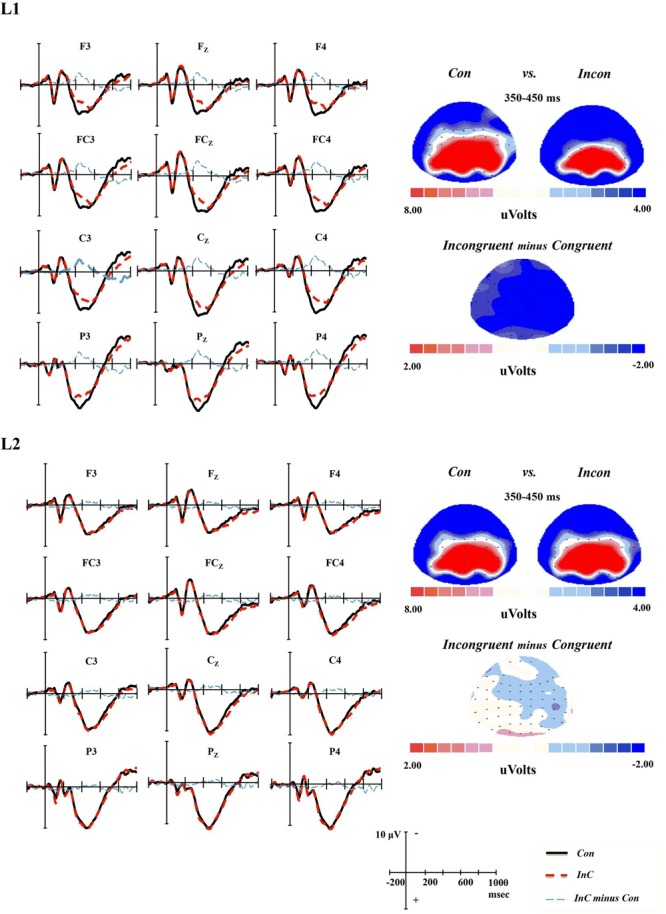
**Grand-average ERPs and topographical maps for the N350–550 component.** Grand-average ERPs at all 12 electrodes for congruent (Con) emotion words (black line) and incongruent (InC) emotion words (red line), as well as the incongruent minus congruent emotion words difference wave (blue line). Topographical maps of the voltage amplitudes for the congruent and incongruent emotion words, as well as the incongruent minus congruent emotion words difference wave at the 350-450 ms time window for L1 and L2 tasks.

The results of repeated measures ANOVAs showed significant congruency effect, larger negativity for incongruent than congruent trials, for both 350-450 ms [*F*(1,17) = 13.98, *p* = 0.002, η_p_^2^ = 0.45] and 450-550 ms [*F*(1,17) = 20.52, *p* < 0.001, η_p_^2^ = 0.55] epochs. For the analysis of average amplitude at 350-450 ms epoch, the two-way interaction of *Congruency* and *Language* was significant [*F*(1,17) = 11.29, *p* = 0.004, η_p_^2^ = 0.40]. Simple effect tests probing this interaction demonstrated that the significant congruency effect was present only in the L1 task [*F*(1,17) = 20.95, *p* < 0.001, η_p_^2^ = 0.55], but not in L2 task [*F*(1,17) = 0.84, *p* = 0.372, η_p_^2^ = 0.05]. In contrast with the results at 350-450 ms epoch, there was no significant *Congruency* × *Language* interaction at 450-550 ms time window (see **Figure 3**).

### Conflict SP

**FIGURE 4:**
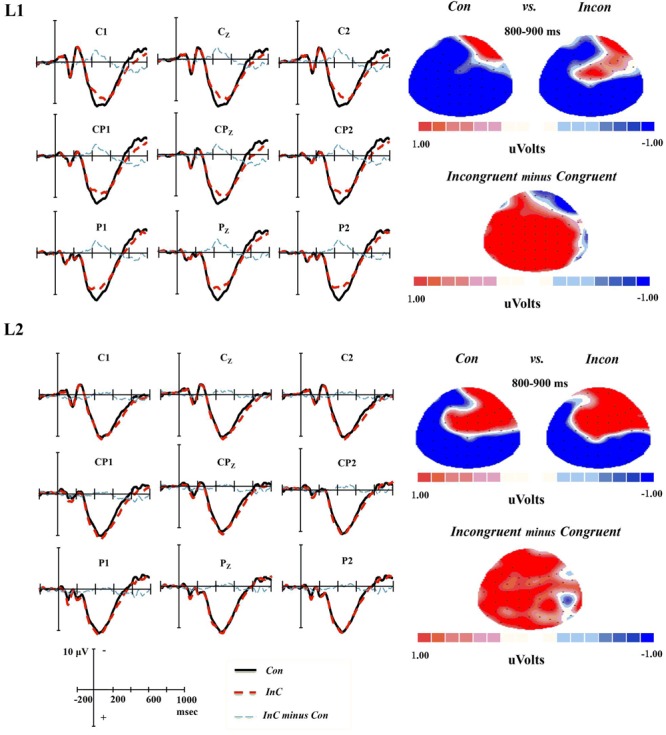
**Grand-average ERPs and topographical maps for the Conflict SP component.** Grand-average ERPs at all nine electrodes for congruent (Con) emotion words (black line) and incongruent (InC) emotion words (red line), as well as the incongruent minus congruent emotion words difference wave (blue line). Topographical maps of the voltage amplitudes for the congruent and incongruent emotion words, as well as the incongruent minus congruent emotion words difference wave at the 800-900 ms time window for L1 and L2 tasks.

A significant congruency effect, increased positivity for incongruent than for congruent trials, was revealed at 700-800 ms [*F*(1,17) = 4.78, *p* = 0.043, η_p_^2^ = 0.22] and 800-900 ms [*F*(1,17) = 5.82, *p* = 0.027, η_p_^2^ = 0.26] epochs. Neither the main effect of language nor the *Congruency* × *Language* interaction reached significance. The results indicated that there was significant congruency effect for conflict SP, but no identifiable language distinction was observed in the effect of this slow potential (see **Figure 4**).

### N250

**FIGURE 5:**
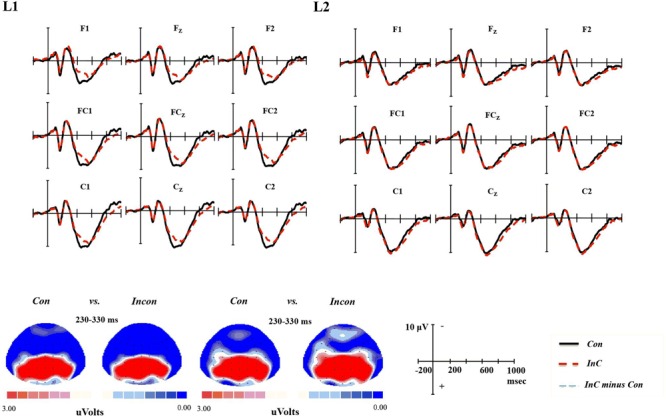
**Grand-average ERPs and topographical maps for the N250 component.** Grand-average ERPs at all nine electrodes for congruent (Con) emotion words (black line) and incongruent (InC) emotion words (red line), and topographical maps of the voltage amplitudes for the congruent and incongruent emotion words at the 230–330 ms time window for L1 and L2 tasks.

A marginally significant *Congruency* × *Language* interaction was reported in the results of repeated measures ANOVAs at the time window 230-350 ms [*F*(1,17) = 4.36, *p* = 0.052 < 0.1, η_p_^2^ = 0.21]. Further analysis of this interaction revealed a significant language effect only in incongruent trials [*F*(1,17) = 8.02, *p* = 0.011, η_p_^2^ = 0.32], with more negativity being observed in the L1 than in the L2 task. Neither the main effect of language nor the main effect of congruency reached significance (see **Figure 5**).

## Discussion

Using behavioral and electrophysiological measures, the present study examined the automatic activation of emotional content of the bilinguals’ L1 and L2 emotion words. The behavioral analysis revealed a significant increase in RT from congruent to incongruent trials in L1 and L2 tasks. For ERP data, we mainly focused on two components (i.e., N250, N350–550) that reflected identifiable language modulation.

### Language Effect in the Behavioral Performance

In the present study, we observed higher accuracy rate and shorter response latencies for congruent than for incongruent stimuli, indicating robust and significant behavioral effect of Stroop interference in our Chinese-English late bilingual population. In line with previous behavioral findings, we interpret this face–word Stroop interference as an automatic processing of L1 and L2 emotion words. The results are consistent with previous L1 studies concerning emotion word processing among participants with different first languages (Chechko et al., 2013; Shen et al., 2013). In our study, there was a face–word Stroop effect in both L1 and L2 tasks, providing novel evidence on the case of a bilingual mind. Furthermore, the results indicated that significantly greater magnitude of interference was found for our late Chinese-English bilinguals’ dominant language (Chinese) as compared to their non-dominant language (English). Consistent with previously identified within-language interference effect in bilingual color-word Stroop tasks (e.g., Mägiste, 1985; MacLeod, 1991; Naylor et al., 2012; Marian et al., 2013), the present finding provides supporting evidence that language proficiency is likely to be a determining factor affecting the magnitude of the Stroop interference effect. The present finding is also reminiscent of the view that there seems to be attenuated sensitivity in the activation of L2 emotional information relative to L1 among late rather than early bilinguals (e.g., Harris et al., 2003, 2006; Dewaele, 2004; Altarriba, 2008; Pavlenko, 2008; Winskel, 2013). The participants in our study started to learn English (L2) rather late in life (AoA = 10.9) and are intermediate learners of English. The dominate language of our participants is their L1 Chinese which is the language of the environment and is used on a daily basis since birth; their self-reported language proficiency of the two languages showed significant difference between their English and Chinese proficiency levels (*p* < 0.001). Accordingly, identifiable differences in the Stroop interference effect were observed in the emotional interference in the two languages.

### Language Effect in the Neural Functioning

Our analysis of N350–550 component revealed significant congruency effect in the L1 task. The finding is reminiscent perfectly of those observed in prior native language studies using the same emotional face–word Stroop paradigm in L1 tasks and is consistent with the notion that the conflict control process begins from approximately 350 ms in the emotional face–word Stroop task (Shen et al., 2013; Xue et al., 2015). Our finding with L1 emotion words is also consistent with N450 results observed in color-word Stroop tasks (e.g., Liotti et al., 2000; Larson et al., 2009; Szücs and Soltész, 2010; Naylor et al., 2012). Different from the L1 task, a robust electrophysiological effect of congruency was not obtained in the L2 task in late Chinese-English bilinguals. Our finding was in agreement with Liu’s (2007) study using color-word Stroop task, which reported statistically insignificant congruency effect in the L2 task. The congruency effect difference across languages seemed to indicate that automatic access to L1 and L2 emotion words differed greatly from each other. On the basis of prior literature, our pattern of results can be attributed to the distinction between L1 and L2 affective processing, as L1 is more sensitive to the emotional content in words than L2.

The time window of N350–550 component was investigated after the onset of emotional and lexical access. The emotional effects, including the EPN and late positive complex (LPC), both begin only after lexical access (Schacht and Sommer, 2009; Palazova et al., 2011, 2013). For instance, using a lexical decision task, Schacht and Sommer (2009) compared the emotion effects in word and face processing. In both domains, they observed an EPN component (peaking around 380 ms) for both emotion words and faces as well as an augmented LPC (peaking around 600 ms) amplitudes to both positive and negative valenced words and to angry faces, appeared at a similar latency. These authors regard the valence effects appearing around 380 ms as post-lexical in nature (Schacht and Sommer, 2009; Palazova et al., 2011; Palazova et al., 2013). This claim was also maintained by our data, with the onsets of valence effects appearing around 350 ms. The N350–550 component also interacted in the N400 semantic effect time window. Similar N400 effects were produced when investigating semantic and emotional information processing (cf. Chen et al., 2013). The present data show that considerable *Congruency* and *Language* interaction was present at 350-450 ms epoch, which appears to reflect different sizes of this negativity across languages. It might imply that the attenuation of N350–550 effect in L2 could best be interpreted as a more pronounced emotional processing advantage in L1 than in L2. With a large number of cognitive studies showing that L2 affective processing may be characterized by attenuated emotionality compared to L1, only the pattern of delayed effect was replicated in previous electrophysiological reports when contrasting L1 and L2 emotion words processing (e.g., Conrad et al., 2011; Opitz and Degner, 2012). Conrad et al. (2011) investigated the neural correlates of emotion effects in L1 and L2 emotion word processing with visual lexical decision task among German-Spanish bilinguals; the results suggested that the amplitudes for respective effects of L1 and L2 emotion words did not qualitatively differ, though EPN emotion effects shifted across L1 and L2 processing about 50-100 ms. In a similar vein, employing a lexical monitoring task, a variant of the lexical decision task, Opitz and Degner (2012) investigated the affective valence of L1 and L2 emotion words among German-French and French-German bilinguals; they found the amplitude of EPN did not differ significantly between L1 and L2, though a delayed EPN effect for L2 was observed. Conrad et al. (2011) viewed the latency shift of EPN emotion effects across L1 and L2 processing as a rather generally delayed L2 visual word processing rather than qualitative differences in the activation of emotional content between L1 and L2 emotion words. The attenuated N350–550 effect in L2 observed in the present study, therefore, make a strong case supporting the claim that the activation or access to L2 emotion words is less automatic and efficient, thus providing supporting evidence for the theoretical proposals assuming attenuated emotionality of L2 processing. As opposed to previous investigations, the emotional face–word Stroop task in the current work provides an experimental approach to examine the affective incompatibility between face emotion and word emotion, thus shedding an interesting light on the issue of affective processing in L1 and L2.

The differences in the N350–550 effect between L1 and L2 might be attributed to the AoA and language proficiency of Chinese-English bilinguals’ relative proficiency of their two languages, as is also mentioned earlier for the behavioral congruency effect. Harris (2004) and Harris et al. (2006) have proposed the emotional contexts of learning theory suggesting that AoA and the language proficiency level may account for the differences in emotionality of L1 and L2 in bilingual speakers. It makes sense that a language learned early in childhood is more deeply coded and will carry strong emotional resonances (Sutton et al., 2007; Caldwell-Harris, 2014) as compared to a weaker association between L2 word and its emotional context (Opitz and Degner, 2012).

Hence, it is plausible that L1 would show an emotionality advantage over L2 for late bilinguals. Tzelgov et al. (1990) and Eilola et al. (2007) proposed that interference in emotional or taboo Stroop tasks may be attributed to bilinguals’ level of relative language proficiency. We consider that this proposal is also in agreement with our finding. As intermediate learners of English who study the target language primarily in a classroom setting and foreign language environment, there exists a great gap between the language proficiency levels of their L1 and L2, thus results in an emotionality disadvantage for L2.

In addition, it is important to note the inconsistency between ERP and behavioral data, i.e. robust behavioral effect of face–word Stroop interference was not accompanied with significant congruency effect at 350-550 ms epoch in the L2 task. Previous research with emotional face–word paradigm, such as Shen et al. (2013), suggested that N350–550 might be associated with the response selection process. Their conclusion was based on the results of correlational analysis that the amplitude of N350–550 covaried with the magnitude of RT. However, our results in L1 indicated that the participants’ Stroop effect failed to correlate with the averaged amplitude at either 350-450 ms (Pearson’s *r* = 0.22, *p* = 0.38) or 450-550 ms interval (Pearson’s *r* = 0.18, *p* = 0.47). Therefore, we propose that the N350–550 component observed in the present study might not be simply associated with conflict resolution as stated in Shen et al. (2013), but a phase awaiting further clarification in the process of conflict processing. This augment remains speculative, and more empirical work is required to further elucidate this point. Interestingly, the same contradictory picture was also observed in the case of Chinese-English bilinguals performing color-word Stroop task (cf. Liu, 2007). Clearly, more empirical studies on the emotionality of L1 and L2 emotion word employing a larger set of word stimuli (e.g., positive, negative, neutral) with participants of different language background, are needed.

For the N250 (230–330 ms, slight medial-lateralization with a fontal maximum) that generally reflects initial stages of orthographic decoding (Ruz and Nobre, 2008; Zhang et al., 2013; Wang and Bastiaansen, 2014), our data show significant *Language* effect only in incongruent trials, with more negativity being observed in the L1 than the L2 task. Ruz and Nobre (2008) observed larger amplitude when the word form information was attended to than when not attended to, suggesting that the amplitude of N250 was modulated by attention. According to this account, the present data indicate that L1 emotion words are more attended to as opposed to L2 emotion words in conflict condition. In other words, more attention is allocated or directed to L1 emotion word than L2. The ability of focusing on task-relevant information and suppressing task-irrelevant distraction—a kind of conflict-driven executive control—plays a critical role in human cognition and behavior (Yang et al., 2015), and this conflict control is a crucial aspect of the executive control mechanism (Zhu et al., 2010). Accordingly, the present data could be interpreted as a consequence of conflict control process which enhances task-relevant information (face emotion) and suppresses task-irrelevant information (L1 or L2 emotion word). The N250 data could also be attributed to the difference in the automatic activation of L1 and L2. Previous studies have often characterized L1 reading as automatic and L2 reading as conscious, controlled and effortful which was slower than L1 reading in general (Favreau and Segalowitz, 1983). Some have interpreted such language distinction as attenuated sensitivity in orthographic and syntactic information for L2 (e.g., Favreau et al., 1980). Our finding concerning N250 has lent support to this notion. Employing the emotional face–word Stroop task with emotion words in task-irrelevant domain enabled us to further explore the reduced sensitivity for L2 emotion word processing at word-form level.

The present study also informs a popular model of bilingual language memory and processing—the revised hierarchical model proposed by Kroll and Stewart (1994). According to this model, the conceptual representation of L1 and L2 is shared while their lexical representation is organized in separate stores. The model proposes that L1 words have stronger links with the conceptual system, as compared to L2 lexical stores. Based on the notions of revised hierarchical model, L1 could produce a larger interference effect and is capable of activating L1 word meanings faster and more automatically as compared to L2 in late bilinguals (Eilola et al., 2007), which reliably predicts less emotional Stroop interference and reduced N350–550 effect in the L2 task in our bilingual population.

Our behavioral data seem to suggest that the Chinese words have an earlier impact on performance than the English words, but the behavioral dynamics of the time course of the effect are not so clear. The issue of determining whether the difference in congruency effect is due to an overall difference in size, or earlier activation of the irrelevant dimension for L1 or L2 processing, is worth further investigation. Further study can investigate this issue by examining the behavioral correlates of L1 and L2 emotion words with distributional analyses of the data (cf. Pratte et al., 2010). The present study examins the processing of written emotion words in task-irrelevant processing, but the reliance on written word stimuli may be a disadvantage since isolated words cannot replace naturalistic interaction. In addition, the ERP effects of the font size and type for L1 and L2 words should be taken into consideration. In this regard, Bayer et al. (2012) found that larger font size leads to an increase of early emotion effects in ERPs for written words. Font size of L1 and L2 words was well-controlled in our study. However, the two languages differ substantially from each other, with one being alphabetic language (English) and the other logographic language (Chinese). The font type of the two languages may have an effect on the L1 and L2 emotion words processing. Further study can be conducted to investigate the ERP effects of font type on the processing of emotionally charged lexical entries between alphabetic and logographic languages.

## Conclusion

We examined the behavioral and neural correlates of task-irrelevant emotion words in late Chinese-English bilinguals. Our study represents the first known attempt to tap into the automaticity of bilingual emotion word processing in a modified Stroop task (i.e., a emotional face–word Stroop manual task) with electrophysiological measurements. In the current study, there was significant face–word Stroop effect in both L1 and L2 tasks, and a larger magnitude of interference effect was found in a bilingual’s dominant language. Our N350–550 data corroborate previous reports, reflecting considerably larger N350–550 amplitude for incongruent than for congruent trials in L1 task. With regard to N250 data, different emotionality between L1 and L2 may influence the emotional conflict control process and thereby impacts the attention allocated to word-form processing in the two languages. Our results are consistent with the view that L2 is less automatic to the activation of emotional content relative to L1, and thus lend support to the revised hierarchical model.

## Author Contributions

LF conceived and designed the work that led to the submission as well as reviewed the manuscript critically; QX performed the experiment, analyzed data, and reviewed the manuscript critically; XW drafted the manuscript; FZ advised on the study and reviewed the manuscript; YY advised on the study and revised the manuscript; XL reviewed and edited the manuscript.

## Conflict of Interest Statement

The authors declare that the research was conducted in the absence of any commercial or financial relationships that could be construed as a potential conflict of interest.

## Footnotes

1 http://www.natcorp.ox.ac.uk/
